# Editorial: Insights in developmental endocrinology: 2021

**DOI:** 10.3389/fendo.2023.1129090

**Published:** 2023-02-02

**Authors:** Lawrence M. Nelson

**Affiliations:** Digital Women's Health Initiative, Mary Elizabeth Conover Foundation, Falls Church, Virginia, United States

**Keywords:** developmental endocrinology, polycystic ovary syndrome, placenta, macrosomia, hypoxia recovery, primary ovarian insufficiency (POI)

Developmental endocrinology research is exciting; the subject is full of intriguing biographical history (1). I first heard of “*Developmental Endocrinology*” in 1986 when I began to work at the USA National Institutes of Health, Bethesda, Maryland. My work was as a research fellow in the Developmental Endocrinology Branch. D. Lynn Loriaux, MD, Ph.D., served as the Branch Chief and Institute Clinical Director. What a rich academic environment he provided. The Branch comprised basic laboratory scientists and world leaders in clinical research in endocrinology - pediatric, medical, and reproductive.

Notably, the long-term funding provided by the NIH Intramural Research Program permitted investigators to conduct in-depth studies of rare disorders, sometimes over several decades. For example, this funding model permitted me to build a team to research the rare disorder of Primary Ovarian Insufficiency for the next 30 years. More about this in due course.

This Research Topic reports on new insights, challenges, and future perspectives across a broad range of critical biological issues. For example, we learn about the cellular response to hypoxia in developing zebrafish, exosomal RNAs in pregnant women with diabetes, the evolution of placental endocrinology, ultrasound criteria of polycystic ovary syndrome, and research on the rare disorder Primary Ovarian Insufficiency (POI).

Zasu et al. study oxygen deprivation, which induces multiple changes at the cellular and organismal levels, and its re-supply also brings another special physiological status. This research team investigated the effects of hypoxia/re-oxygenation on embryonic growth using the zebrafish model: hypoxia slows embryonic growth, but re-oxygenation induces growth spurt or catch-up growth. Their results indicate that redox signaling alters IGF/Igf signaling to facilitate hypoxia/re-oxygenation-induced embryonic growth compensation.

Yuan et al. studied exosomes, cell-derived vesicles in many biological fluids. For example, exosomal RNAs in cord blood may allow intercellular communication between mother and fetus. Notably, their results show the aberrant expression of exosomal RNAs in the cord blood of patients with gestational diabetes mellitus (GDM).

Furthermore, the findings may highlight essential aspects of exosomal RNAs in the peripheral blood of women with GDM.

Carter, in a comprehensive report, brings us up to date on the endocrinology of placental evolution. The human placenta secretes a variety of hormones, some of them in large amounts. Their effects on maternal physiology, including the immune system, are poorly understood. The review informs on the evolution of placental hormones involved in the recognition and maintenance of pregnancy, in maternal adaptations to pregnancy and lactation, and in facilitating immune tolerance of the fetal semiallograft. Knowledge gained from laboratory and domesticated mammals can translate to a better understanding of human placental endocrinology if viewed in an evolutionary context.

Giménez-Peralta et al. set out to define ultrasound criteria to define the best indicator of developing metabolic and endocrine changes in women with polycystic ovary syndrome (PCOS).

Their multicenter cross-sectional controlled study included 200 women with PCOS and normal controls. The main finding in this study points toward a different ultrasound criterion—23 or more follicles of any size in at least one ovary. An ovarian ultrasound examination with 23 or more follicles of any size in any ovaries constitutes a powerful tool to diagnose PCOS and associate it with metabolic–endocrine processes such as hyperandrogenism and insulin resistance.

Zhang et al. conduct a bibliometric analysis of Primary Ovarian Insufficiency (POI), a heterogeneous disease with diverse clinical phenotypes and etiologies. They identify research hotspots and trends in POI. The USA was dominant in the field of POI in terms of the number of publications, average citations per item, and h-index. The research hotspots in POI are mainly pathogenesis and treatment, including genetic mutation, hormone therapy, and fertility preservation. As shown in **Figure 1**, there has been a dramatic increase in the number of yearly publications on POI in the past two decades.

**Figure 1:**
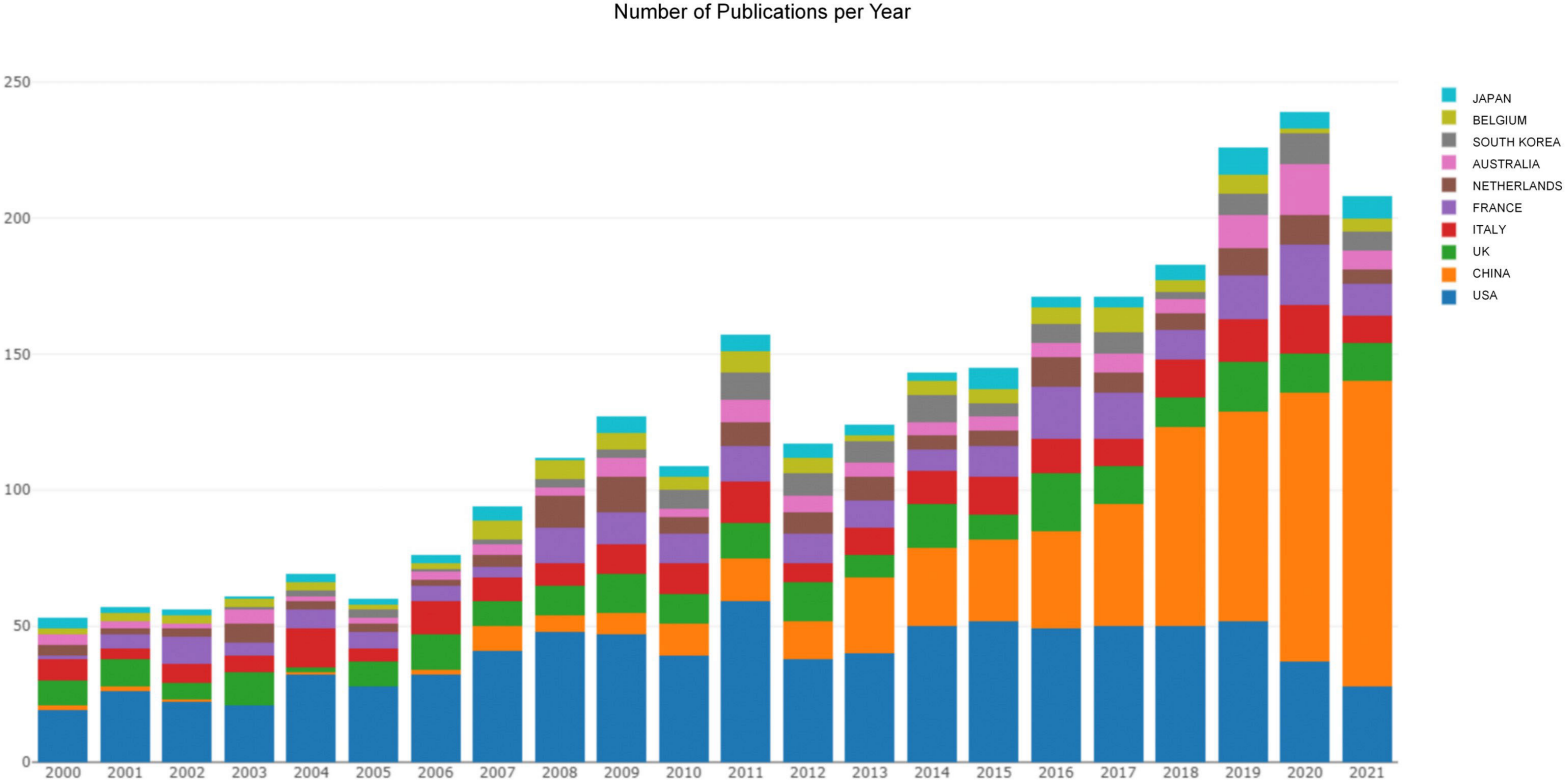
The publishing performance of countries. The yearly production trend per country in the field of POI published from 2000 to 2021. (Zhang et al.).

There have been other notable studies that have analyzed research in POI. For example, Deng et al. (2) also found the USA produced the most influence in this field. Tong et al. (3) found that the USA, Shanghai Jiao Tong University, and *Human Reproduction* were the most productive country, organization, and journal, respectively. Harvard University was the organization with the highest citation. *Fertility and Sterility and* the NIH POI research team led by LM Nelson were the most influential journal and research team, respectively. The finding which makes the NIH POI research team the most influential group advancing the field of POI research supports the NIH Intramural Research Program method of supporting investigative teams with long-term funding, sometimes several decades. In conducting research on rare disorders the most challenging aspect is to have enough patients participate in the research. There is a need for a new approach to POI which is sustainable over the longer term, global in scope, and based in a cloud based digital enterprise. Conover Foundation has initiated such an effort which they have termed 28 Days. (Ref - https://my28days.org/).

## Author contributions

The author confirms being the sole contributor of this work and has approved it for publication.
